# Hyperbaric Oxygen Exposure Reduces Age-Related Decrease in Oxidative Capacity of the Tibialis Anterior Muscle in Mice

**DOI:** 10.4061/2010/824763

**Published:** 2010-01-19

**Authors:** Takahiro Nishizaka, Fumiko Nagatomo, Hidemi Fujino, Tomoko Nomura, Tomohiko Sano, Kazuhiko Higuchi, Isao Takeda, Akihiko Ishihara

**Affiliations:** ^1^Beauty Care Research Laboratories, Kao Corporation, Tokyo 131-8501, Japan; ^2^Laboratory of Neurochemistry, Graduate School of Human and Environmental Studies, Kyoto University, Sakyo-ku, Kyoto 606-8501, Japan; ^3^Division of Rehabilitation Sciences, Kobe University Graduate School of Health Sciences, Kobe 654-0142, Japan; ^4^Biological Science Laboratories, Kao Corporation, Tochigi 321-3497, Japan; ^5^Department of Physical Therapy, Faculty of Health Care Science, Himeji Dokkyo University, Himeji 670-8524, Japan

## Abstract

The effects of exposure to hyperbaric oxygen on the oxidative capacity of the skeletal muscles in mice at different ages were investigated. We exposed 5-, 34-, 55-, and 88-week-old mice to 36% oxygen at 950 mmHg for 6 hours per day for 2 weeks. The activities of succinate dehydrogenase (SDH), which is a mitochondrial marker enzyme, of the tibialis anterior muscle in hyperbaric mice were compared with those in age-matched mice under normobaric conditions (21% oxygen at 760 mmHg). Furthermore, the SDH activities of type IIA and type IIB fibers in the muscle were determined using quantitative histochemical analysis. The SDH activity of the muscle in normobaric mice decreased with age. Similar results were observed in both type IIA and type IIB fibers in the muscle. The decrease in the SDH activity of the muscle was reduced in hyperbaric mice at 57 and 90 weeks. The decreased SDH activities of type IIA and type IIB fibers were reduced in hyperbaric mice at 90 weeks and at 57 and 90 weeks, respectively. We conclude that exposure to hyperbaric oxygen used in this study reduces the age-related decrease in the oxidative capacity of skeletal muscles.

## 1. Introduction

A reduction in skeletal muscle mass is one of the most striking features of the aging process. Previous studies [1–3] have indicated that this reduction is due to decreases in the number and volume of individual fibers in skeletal muscles. Mammalian skeletal muscles consist of different sizes and types of fibers, for example, slow-twitch type I and fast-twitch type II fibers [4, 5]. A reduction in the number and volume of type II fibers in skeletal muscles of rats can be observed in the initial stages of the aging process [6, 7]. These changes in type II fibers are considered to be due to a transition of fiber types from type II to type I, selective loss and atrophy of type II fibers, and/or degeneration in the neuromuscular junction, which are induced by age-related disuse of type II fibers. A decrease in the number and volume of both type I and type II fibers in skeletal muscles of rats can be observed in the late stages of the aging process [6–8]. These changes in type I and type II fibers are closely related to the loss and degeneration of spinal motoneurons innervating those fibers in skeletal muscles. Furthermore, a decrease in the oxidative enzyme activity of skeletal muscles in rats was observed with increasing age [9–11].

An elevation in atmospheric pressure accompanied by an increase in oxygen concentration enhances the partial pressure of oxygen and increases the concentration of dissolved oxygen in the plasma. An increase in both atmospheric pressure and oxygen concentration enhances oxidative enzyme activity in mitochondria and consequently increases the oxidative metabolism in cells and tissues [12]; thus, it is expected that exposure to hyperbaric oxygen facilitates the turnover of oxidative metabolism, particularly of pathways in the mitochondrial TCA cycle, thereby reducing the age-related decrease in the oxidative enzyme activity of muscle fibers. We determined that a pressure of 960 mmHg and an oxygen concentration of 36% are required for obtaining effective responses with regard to oxidative metabolism [12, 13]. This study examined the oxidative capacity of the tibialis anterior muscle in mice at different ages, which were exposed to 36% oxygen at 950 mmHg. Furthermore, the cross-sectional areas and oxidative enzyme activities of fibers, which were type-defined by ATPase activity, in the muscle of mice were determined using quantitative histochemical analysis.

## 2. Materials and Methods

All experimental procedures, including animal care, were conducted in accordance with the Guide for the Care and Use of Laboratory Animals of the Japanese Physiological Society. This study was also approved by the Institutional Animal Care Committee at Kyoto University.

### 2.1. Animal Care and Treatment

We used 5-, 34-, 55-, and 88-week-old female mice in this study. The mice (the hyperbaric group; *n* = 6 in each age group) were exposed to hyperbaric conditions (950 mmHg) with a high oxygen concentration (36%), which were automatically maintained by a computer-assisted system, in a hyperbaric chamber for 6 hours (1100–1700) and were placed under normobaric conditions (21% oxygen at 760 mmHg) for 18 hours (1700–1100), while other mice (the normobaric group; *n* = 6 in each age group) were placed in a hyperbaric chamber under normobaric conditions for 24 hours. The hyperbaric chamber was 90 cm in length and 80 cm in diameter; thus, it could simultaneously house a number of rats (up to 20 cages).

All mice were individually housed in same-sized cages in a room maintained under controlled 12-hour light/dark cycles (lights switched off from 2000 to 0800) at a temperature of 22 ± 2°C with a relative humidity of 45%–65%. Food and water were provided *ad libitum* to all mice.

### 2.2. Tissue Procedures

After 2 weeks of exposure to hyperbaric oxygen, the mice in the normobaric and hyperbaric groups were anesthetized by an intraperitoneal injection of sodium pentobarbital (50 mg/kg body weight). The tibialis anterior muscles from both hind limbs were removed and cleaned of excess fat and connective tissue. Thereafter, the mice were sacrificed by an overdose of sodium pentobarbital.

The tibialis anterior muscles of the right side were quickly frozen in liquid nitrogen for measurement of succinate dehydrogenase (SDH) activity. The SDH activity was determined according to the method of Cooperstein et al. [14]. Briefly, the muscles were homogenized using a glass tissue homogenizer with 5 volumes of ice-cold 0.3 M phosphate buffer, pH 7.4. Sodium succinate was added to yield a final concentration of 17 mM. The final concentrations of the components of the reaction mixture were as follows: sodium succinate 17 *μ*M, sodium cyanide 1 mM, aluminum chloride 0.4 mM, and calcium chloride 0.4 mM. This reaction mixture was transferred to the spectrophotometer and the reduction of cytochrome *c* was followed by observing the increase in extinction at 550 nm. The SDH activity was calculated from the ferricytochrome *c* concentration and protein content.

The tibialis anterior muscles of the left side were pinned on a cork at their *in vivo *length and quickly frozen in isopentane cooled with liquid nitrogen. The mid-portion of the muscle was mounted on a specimen chuck using a Tissue Tek OCT Compound (Sakura Finetechnical, Tokyo, Japan). Serial transverse sections (10-*μ*m thickness) of the muscle on the chuck were cut in a cryostat maintained at −20°C. The serial sections were brought to room temperature, air-dried for 30 minutes, and incubated for ATPase activity following acid preincubation and for SDH activity [15, 16].

**Figure 1:**
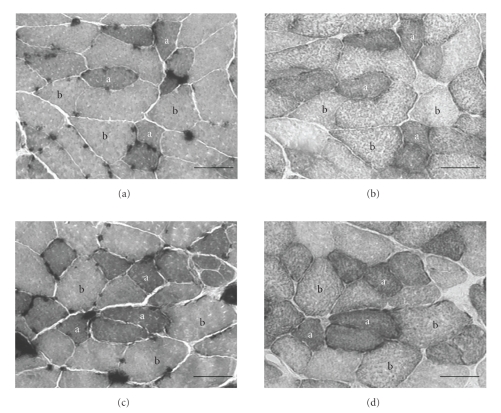
Serial transverse sections of the tibialis anterior muscles in the normobaric ((a) and (b)) and hyperbaric ((c) and (d)) mice at 90 weeks. (a) and (c), stained for ATPase activity following preincubation at pH 4.5; (b) and (d), stained for succinate dehydrogenase activity. a: type IIA; b: type IIB. Scale bar  = 50 *μ*m.

The ATPase activity was determined by the following procedures: (1) preincubation for 5 minutes at room temperature in 50 mM sodium acetate and 30 mM sodium barbital in distilled water, adjusted to pH 4.5 with HCl; (2) washing in 5 changes of distilled water; (3) incubation for 45 minutes at 37°C in 2.8 mM ATP, 50 mM CaCl_2_, and 75 mM NaCl in distilled water, adjusted to pH 9.4 with NaOH; (4) washing in 5 changes of distilled water; (5) immersion for 3 minutes in 1% CaCl_2_; (6) washing in 5 changes of distilled water; (7) immersion for 3 minutes in 2% CoCl_2_; (8) washing in 5 changes of distilled water; (9) immersion for 1 minutes in 1% (NH_4_)_2_S; (10) washing in 5 changes of distilled water; (11) dehydration in a graded series of ethanol, passed through xylene, and then cover slipped (Figure 1). Classification into two fiber types was based on staining intensities for ATPase activity: type IIA (positive intensity) and type IIB (negative intensity) [17].

The SDH activity was determined by incubation in a medium containing 0.9 mM 1-methoxyphenazine methylsulfate, 1.5 mM nitroblue tetrazolium, 5.6 mM ethylenediaminetetraacetic acid disodium salt, and 48 mM succinate disodium salt (pH 7.6) in 100 mM phosphate buffer. The incubation time was 10 minutes; the changes in staining intensity in response to incubation reached a plateau after 10 minutes. The reaction was stopped by multiple washings with distilled water, dehydrated in a graded series of ethanol, passed through xylene, and cover slipped (Figure 1).

The cross-sectional areas and SDH activities from approximately 300 fibers, which were type-defined by ATPase activity, in the central region of the muscle section were measured by tracing the outline of a fiber and stored in a computer-assisted image processing system (Neuroimaging System, Kyoto, Japan) [18, 19]. The images were digitized as gray-level pictures. Each pixel was quantified as one of 256 gray levels that were then automatically converted to optical density (OD). A gray level of zero was equivalent to 100% transmission of light and that of 255 was equivalent of 0% transmission of light. The mean OD value of all pixels within a fiber was determined using a calibration tablet that had 21 gradient density steps and corresponding diffused density values.

### 2.3. Statistical Analyses

The data were expressed as mean and standard deviation. One-way analysis of variance was used to evaluate the age-related changes. When the differences were found to be significant, further comparisons were made by performing *post hoc* tests. The differences between the normobaric and age-matched hyperbaric groups were determined by using the *t*-test. A probability level of 0.05 was considered to be statistically significant.

## 3. Results

### 3.1. Body Weight

**Figure 2:**
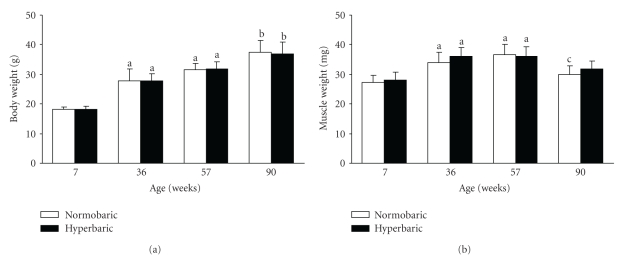
Body weights (a) and tibialis anterior muscle weights (b) of the normobaric and hyperbaric groups at different ages. Data are represented as the mean and standard deviation determined from six animals. The mice in the hyperbaric group were exposed to 36% oxygen at 950 mmHg for 6 hours per day for 2 weeks. ^a^ *P* < .05 compared with the corresponding group at 7 weeks; ^b^ *P* < .05 compared with the corresponding groups at 7, 36, and 57 weeks; ^c^ *P* < .05 compared with the corresponding group at 57 weeks.

An age-related increase in body weight was observed in the normobaric groups; the body weights at 36 and 57 weeks were greater than that at 7 weeks, and the body weight at 90 weeks was the greatest among the groups (Figure 2(a)). These results were similar in the hyperbaric groups.

There were no differences in body weight between the normobaric and age-matched hyperbaric groups, irrespective of the age.

### 3.2. Tibialis Anterior Muscle Weight

The muscle weights of the normobaric groups at 36 and 57 weeks were greater than that at 7 weeks (Figure 2(b)). These results were similar in the hyperbaric groups. The muscle weight of the normobaric group at 90 weeks was lower than that at 57 weeks.

There were no differences in muscle weight between the normobaric and age-matched hyperbaric groups, irrespective of the age.

### 3.3. SDH Activity of the Tibialis Anterior Muscle

**Figure 3:**
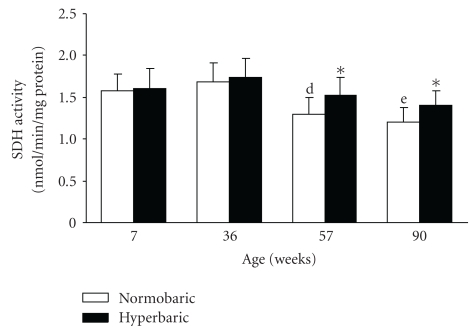
Succinate dehydrogenase activities of the tibialis anterior muscles of the normobaric and hyperbaric groups at different ages. Data are represented as the mean and standard deviation determined from six animals. The mice in the hyperbaric group were exposed to 36% oxygen at 950 mmHg for 6 hours per day for 2 weeks. SDH: succinate dehydrogenase. ^d^ *P* < .05 compared with the corresponding group at 36 weeks; ^e^ *P* < .05 compared with the corresponding groups at 7 and 36 weeks; *P < .05 compared with the age-matched normobaric group.

An age-related decrease in SDH activity was observed in the normobaric groups; the SDH activities of the muscle at 57 and 90 weeks were lower than that at 36 weeks and those at 7 and 36 weeks, respectively (Figure 3). There were no differences in SDH activity of the muscle among the hyperbaric groups, irrespective of the age.

The SDH activity of the muscle in the hyperbaric group at 57 and 90 weeks was greater than that in the age-matched normobaric group.

### 3.4. Fiber Cross-Sectional Area in the Tibialis Anterior Muscle

**Figure 4:**
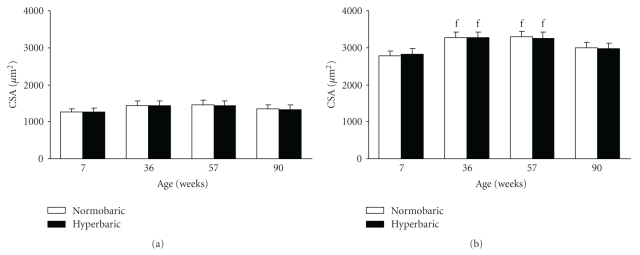
Cross-sectional areas of type IIA (a) and type IIB (b) fibers in the tibialis anterior muscles of the normobaric and hyperbaric groups at different ages. Data are represented as the mean and standard deviation determined from six animals. The mice in the hyperbaric group were exposed to 36% oxygen at 950 mmHg for 6 hours per day for 2 weeks. CSA: cross-sectional area. ^f^ *P* < .05 compared with the corresponding groups at 7 and 90 weeks.

There were no differences in cross-sectional area of type IIA fibers among the normobaric groups, irrespective of the age (Figure 4(a)). These results were similar in the hyperbaric groups.

There were no differences in cross-sectional area of type IIA fibers between the normobaric and age-matched hyperbaric groups, irrespective of the age.

The cross-sectional areas of type IIB fibers in the normobaric groups at 36 and 57 weeks were greater than those at 7 and 90 weeks (Figure 4(b)). These results were similar in the hyperbaric groups.

There were no differences in cross-sectional area of type IIB fibers between the normobaric and age-matched hyperbaric groups, irrespective of the age.

### 3.5. Fiber SDH Activity in the Tibialis Anterior Muscle

**Figure 5:**
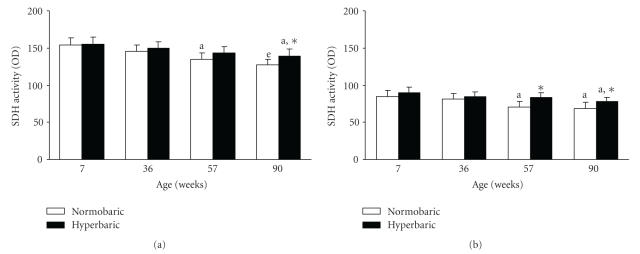
Succinate dehydrogenase activities of type IIA (a) and type IIB (b) fibers in the tibialis anterior muscles of the normobaric and hyperbaric groups at different ages. Data are represented as the mean and standard deviation determined from six animals. The mice in the hyperbaric group were exposed to 36% oxygen at 950 mmHg for 6 hours per day for 2 weeks. SDH: succinate dehydrogenase; OD: optical density. ^a^ *P* < .05 compared with the corresponding group at 7 weeks; ^e^ *P* < .05 compared with the corresponding groups at 7 and 36 weeks; *P < .05 compared with the age-matched normobaric group.

The SDH activity of type IIA fibers in the normobaric group at 57 weeks was lower than that at 7 weeks (Figure 5(a)). The SDH activity of type IIA fibers in the normobaric group at 90 weeks was lower than those at 7 and 36 weeks. The SDH activity of type IIA fibers in the hyperbaric group at 90 weeks was lower than that at 7 weeks.

The SDH activity of type IIA fibers in the hyperbaric group at 90 weeks was greater than that in the age-matched normobaric group.

The SDH activities of type IIB fibers in the normobaric groups at 57 and 90 weeks were lower than that at 7 weeks (Figure 5(b)). The SDH activity of type IIB fibers in the hyperbaric group at 90 weeks was lower than that at 7 weeks.

The SDH activity of type IIB fibers in the hyperbaric group at 57 and 90 weeks was greater than that of the age-matched hyperbaric group.

## 4. Discussion

An elevation in atmospheric pressure accompanied by high oxygen concentration enhances the partial pressure of oxygen and increases the concentration of dissolved oxygen in the plasma [20, 21]. An increase in both atmospheric pressure and oxygen concentration enhances the mitochondrial oxidative enzyme activity and consequently increases oxidative metabolism in cells and tissues. Furthermore, an increase in atmospheric pressure and oxygen concentration increases carbon dioxide concentration, which in turn facilitates the release of oxygen from hemoglobin and causes the dilation of blood vessels. We designed a hyperbaric chamber for performing the animal experiments [12]; the chamber consisted of an oxygen tank containing an oxygen concentrator and an air compressor, which automatically maintains the elevated atmospheric pressure and oxygen concentration using a computer-assisted system. We determined the optimal atmospheric pressure (950 mmHg) and oxygen concentration (36%) required for obtaining effective responses with regard to oxidative capacity in the neuromuscular system [12].

Our previous study [13] demonstrated that young rats exposed to 36% oxygen at 950 mmHg exhibited greater voluntary running activities than those maintained under normobaric conditions. We also found that oxidative enzyme activities of fibers in the soleus and plantaris muscles and of spinal motoneurons innervating these muscles increased following exposure to hyperbaric oxygen [13]. These findings suggest that the adaptation of neuromuscular units to hyperbaric oxygen enhances the oxidative capacity in muscle fibers and motoneurons, which promotes the function of the neuromuscular units. Furthermore, our previous studies [22, 23] revealed that exposure to 36% oxygen at 950 mmHg inhibited the growth-related increase in blood glucose levels of type 2 diabetic rats and in blood pressure levels of spontaneously hypertensive rats. Exposure to hyperbaric oxygen inhibited both the growth-related transition of fiber types from high to low oxidative and the decrease in oxidative enzyme activity of fibers in the soleus and plantaris muscles of type 2 diabetic rats [24, 25]. It is suggest that exposure to hyperbaric oxygen reduces the age-related decrease in the oxidative capacity of skeletal muscles, because exposure to hyperbaric oxygen facilitates the turnover of oxidative metabolism, particularly of pathways in the mitochondrial TCA cycle.

Exercise is believed to be effective in maintaining and improving oxidative metabolism in cells and tissues. Our previous study [26] observed that exercise is effective for the prevention of a decrease in the oxidative enzyme activity of type I and type II fibers in the soleus muscles of rats, which was induced by unloading. Furthermore, our previous study [27] found that running exercises served to inhibit the growth-related transition of fiber types from high to low oxidative in the soleus muscle of rats with type 2 diabetes, although this inhibition was observed only in rats that ran more than 7 km per day.

Atrophy, loss, and decreased oxidative enzyme activity of fibers in skeletal muscles have been observed with increasing age [6, 7]. Muscle atrophy in old rats is associated with a decrease in activity levels of certain enzymes involved in oxidative metabolism [10]. These changes in skeletal muscles of rats in the initial stages of aging (60–65 weeks) are considered to be due to the age-related disuse of skeletal muscles, which results in the lowering of oxidative capacity of individual fibers. A previous study [9] observed that 96-week-old rats retained the capacity to increase the oxidative enzyme activity and mitochondrial density of skeletal muscles in response to endurance exercises. Furthermore, our previous study [28] observed that voluntary running exercises prevented atrophy of type II fibers as well as the decrease in oxidative enzyme activity of type I and type II fibers in rats in the initial stages of aging (65 weeks). Therefore, it is expected that a reduction in the decrease of oxidative metabolism in skeletal muscles, which was induced by exposure to hyperbaric oxygen as well as by aerobic exercise, should treat fiber atrophy and the decrease in oxidative capacity of skeletal muscles during the initial stages of the aging process.

We classified fibers in the tibialis anterior muscles of mice into two types on the basis of staining intensities for the ATPase activity: type IIA and type IIB. In normobaric mice, type IIA fibers were smaller than type IIB fibers, irrespective of the age (Figure 4). Type IIA fibers are more effective in supplying oxygen and nutrients for oxidative metabolism from capillaries, which are located close to the membrane, because of their small sizes. These indicate that type IIA fibers can work at a relatively low intensity and have more prolonged activities than do type IIB fibers. In this study, a reduction in cross-sectional area of type IIB fibers (Figure 4(b)), but not type IIA fibers (Figure 4(a)), was observed at 90 weeks. Low-intensity and prolonged activities, which are performed presumably using type IIA fibers, continue during increasing age, while high-intensity and short activities, which are performed presumably using type IIB fibers, decrease with increasing age. These indicate that type IIB fibers become less active with increasing age and, therefore, facilitate disuse-induced atrophy as observed in Figure 4(b). In this study, there were no differences in cross-sectional area of type IIA or type IIB fibers between the normobaric and age-related hyperbaric mice (Figure 4). Therefore, exposure to hyperbaric oxygen had no effect on fiber cross-sectional area in the muscle. This view does not match our expectations and is inconsistent with the findings observed in relation to exercise [28].

Exposure to hyperbaric oxygen reduced the age-related decrease in the oxidative enzyme activity of the tibialis anterior muscle (Figure 3). Similarly, exposure to hyperbaric oxygen reduced the oxidative enzyme activity of type IIB fibers in the muscle at 57 weeks (initial stage of aging) and those of type IIA and type IIB fibers at 90 weeks (middle to late stages of aging) (Figure 5). The changes in the oxidative enzyme activity of the tibialis anterior muscle by exposure to hyperbaric oxygen corresponded well with that of muscle fibers. We conclude that exposure to hyperbaric oxygen used in this study reduced the age-related decrease in the oxidative capacity of skeletal muscles because of the increased oxidative metabolism in cells and tissues.

## References

[1] Carry MR, Horan SE, Reed SM, Farrell RV (1993). Structure, innervation, and age-associated changes of mouse forearm muscles. *Anatomical Record*.

[2] Holloszy JO, Chen M, Cartee GD, Young JC (1991). Skeletal muscle atrophy in old rats: differential changes in the three fiber types. *Mechanisms of Ageing and Development*.

[3] Lexell J (1995). Human aging, muscle mass, and fiber type composition. *Journals of Gerontology. Series A*.

[4] Nakatani T, Nakashima T, Kita T (1999). Succinate dehydrogenase activities of fibers in the rat extensor digitorum longus, soleus, and cardiac muscles. *Archives of Histology and Cytology*.

[5] Nakatani T, Nakashima T, Kita T (2000). Cell size and oxidative enzyme activity of different types of fibers in different regions of the rat plantaris and tibialis anterior muscles. *Japanese Journal of Physiology*.

[6] Ishihara A, Araki H (1988). Effects of age on the number and histochemical properties of muscle fibers and motoneurons in the rat extensor digitorum longus muscle. *Mechanisms of Ageing and Development*.

[7] Ishihara A, Naitoh H, Katsuta S (1987). Effects of ageing on the total number of muscle fibers and motoneurons of the tibialis anterior and soleus muscles in the rat. *Brain Research*.

[8] Hirofuji C, Ishihara A, Roy RR (2000). SDH activity and cell size of tibialis anterior motoneurons and muscle fibers in SAMP6. *NeuroReport*.

[9] Beyer RE, Starnes JW, Edington DW, Lipton RJ, Compton RT, Kwasman MA (1984). Exercise-induced reversal of age-related declines of oxidative reactions, mitochondrial yield, and flavins in skeletal muscle of the rat. *Mechanisms of Ageing and Development*.

[10] Cartee GD, Farrar RP (1987). Muscle respiratory capacity and Vo2 max in identically trained young and old rats. *Journal of Applied Physiology*.

[11] Kovanen V, Suominen H (1987). Effects of age and life-time physical training on fibre composition of slow and fast skeletal muscle in rats. *Pflügers Archiv European Journal of Physiology*.

[12] Ishihara A, Kawano F, Okiura T, Morimatsu F, Ohira Y (2005). Hyperbaric exposure with high oxygen concentration enhances oxidative capacity of neuromuscular units. *Neuroscience Research*.

[13] Matsumoto A, Okiura T, Morimatsu F, Ohira Y, Ishihara A (2007). Effects of hyperbaric exposure with high oxygen concentration on the physical activity of developing rats. *Developmental Neuroscience*.

[14] Cooperstein SJ, Lazarow A, Kurfess NJ (1950). A microspectrophotometric method for the determination of succinic dehydrogenase. *The Journal of Biological Chemistry*.

[15] Ishihara A, Itoh K, Itoh M, Hirofuji C (2000). Effect of hypobaric hypoxia on rat soleus muscle fibers and their innervating motoneurons: a review. *Japanese Journal of Physiology*.

[16] Nakatani T, Nakashima T, Kita T, Ishihara A (2003). Cell size and oxidative enzyme activity of type-identified fibers in rat hindlimb muscles: a review. *Acta Histochemica et Cytochemica*.

[17] Hirofuji C, Nakatani T, Ishihara A (2000). Cell size and succinate dehydrogenase activity of different types of fibers in different regions of the tibialis anterior muscle in mice and rats. *Acta Histochemica et Cytochemica*.

[18] Nagatomo F, Gu N, Fujino H, Takeda I, Tsuda K, Ishihara A (2009). Skeletal muscle characteristics of rats with obesity, diabetes, hypertension, and hyperlipidemia. *Journal of Atherosclerosis and Thrombosis*.

[19] Nagatomo F, Ishihara A, Ohira Y (2009). Effects of hindlimb unloading at early postnatal growth on cell body size in spinal motoneurons innervating soleus muscle of rats. *International Journal of Developmental Neuroscience*.

[20] Leach RM, Rees PJ, Wilmshurst P (1998). ABC of oxygen: hyperbaric oxygen therapy. *British Medical Journal*.

[21] Tibbles PM, Edelsberg JS (1996). Hyperbaric-oxygen therapy. *The New England Journal of Medicine*.

[22] Nagatomo F, Fujino H, Takeda I,  Ishihara A Effects of hyperbaric oxygenation on blood pressure levels of spontaneously hypertensive rats.

[23] Yasuda K, Aoki N, Adachi T (2006). Hyperbaric exposure with high oxygen concentration inhibits growth-associated increase in the glucose level of diabetic Goto-Kakizaki rats. *Diabetes, Obesity and Metabolism*.

[24] Matsumoto A, Nagatomo F, Yasuda K, Tsuda K, Ishihara A (2007). Hyperbaric exposure with high oxygen concentration improves altered fiber types in the plantaris muscle of diabetic Goto-Kakizaki rats. *Journal of Physiological Sciences*.

[25] Yasuda K, Adachi T, Gu N (2007). Effects of hyperbaric exposure with high oxygen concentration on glucose and insulin levels and skeletal muscle-fiber properties in diabetic rats. *Muscle and Nerve*.

[26] Ishihara A, Kawano F, Ishioka N (2004). Effects of running exercise during recovery from hindlimb unloading on soleus muscle fibers and their spinal motoneurons in rats. *Neuroscience Research*.

[27] Yasuda K, Adachi T, Kikuchi N (2006). Effects of running exercise on fibre-type distribution of soleus and plantaris muscles in diabetic Otsuka Long-Evans Tokushima fatty rats. *Diabetes, Obesity and Metabolism*.

[28] Ishihara A, Taguchi S (1993). Effect of exercise on age-related muscle atrophy. *Neurobiology of Aging*.

